# Prion Fragment Peptides Are Digested with Membrane Type Matrix Metalloproteinases and Acquire Enzyme Resistance through Cu^2+^-Binding

**DOI:** 10.3390/biom4020510

**Published:** 2014-05-08

**Authors:** Aya Kojima, Motomi Konishi, Toshifumi Akizawa

**Affiliations:** 1 Analytical Chemistry, Pharmaceutical Science, Setsunan University, 45-1 Nagaotoge-cho, Hirakata, Osaka 573-0101, Japan; E-Mails: ayak@ims.u-tokyo.ac.jp (A.K.); motomi@pharm.setsunan.ac.jp (M.K.); 2 Division of Cellular and Molecular Biology, Department of Cancer Biology, Institute of Medical Science, The University of Tokyo, 4-6-1 Shirokanedai, Minato-ku, Tokyo 180-8639, Japan

**Keywords:** prion protein, fragment peptide, MT1-MMP, MT3-MMP, MMP-7, Cu^2+^, degradation

## Abstract

Prions are the cause of neurodegenerative disease in humans and other mammals. The structural conversion of the prion protein (PrP) from a normal cellular protein (PrP^C^) to a protease-resistant isoform (PrP^Sc^) is thought to relate to Cu^2+^ binding to histidine residues. In this study, we focused on the membrane-type matrix metalloproteinases (MT-MMPs) such as MT1-MMP and MT3-MMP, which are expressed in the brain as PrP^C^-degrading proteases. We synthesized 21 prion fragment peptides. Each purified peptide was individually incubated with recombinant MT1-MMP or MT3-MMP in the presence or absence of Cu^2+^ and the cleavage sites determined by LC-ESI-MS analysis. Recombinant MMP-7 and human serum (HS) were also tested as control. hPrP61-90, from the octapeptide-repeat region, was cleaved by HS but not by the MMPs tested here. On the other hand, hPrP92-168 from the central region was cleaved by MT1-MMP and MT3-MMP at various sites. These cleavages were inhibited by treatment with Cu^2+^. The C-terminal peptides had higher resistance than the central region. The data obtained from this study suggest that MT-MMPs expressed in the brain might possess PrP^C^-degrading activity.

## 1. Introduction

Prion protein (PrP) is a cell-surface glycoprotein implicated in the pathogenesis of a range of neurodegenerative disorders collectively termed transmissible spongiform encephalopathies, including Creutzfeldt-Jakob disease in humans, bovine spongiform encephalopathy in cows, and chronic wasting disease in deer [1,2,3,4]. Although there is no difference in the primary structure of cellular PrP (PrP^C^) and a pathogenic or scrapie form (PrP^Sc^), spectroscopic studies revealed that PrP^C^ has a high α-helical content whereas PrP^Sc^ is composed primarily of β-sheets [5,6]. There are many hypotheses regarding the conversion of PrP^C^ to PrP^Sc^, but the data suggest that conversion is entirely conformational and involves no amino acid substitutions or deletions, and, thus, supports the protein-only hypothesis [2]. Prion replication requires the conversion of PrP^C^ into PrP^Sc^, where PrP^Sc^ acts as a template and protein X functions as a chaperone [7,8,9].

**Figure 1:**
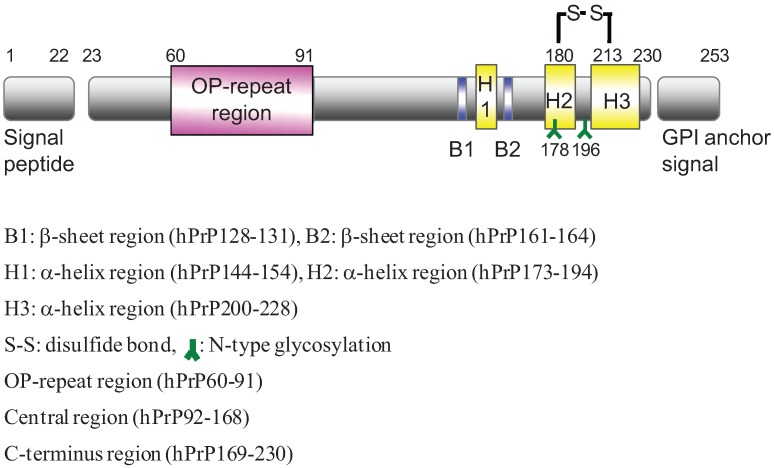
Domain structure of human prion protein (hPrP).

Mature human PrP (hPrP) consists of 253 amino acids (Figure 1). The N-terminal domain, which includes four repeats of the PHGGGWGQ octapeptide, is a flexibly disordered region. In contrast, the C-terminal domain, which includes two a-helices, two glycosylation sites, and a glycosylphosphatidylinositol (GPI) anchor, is a folded region. The middle domain includes two β-sheets and one α-helix [10,11,12]. The hPrP region that spans amino acid residues 106–126 in the middle domain is thought to be responsible for the pathogenic properties of PrP^Sc^, including neurotoxicity, protease resistance, induction of hypertrophy, and promotion of astrocyte proliferation [13,14,15,16,17].

Matrix metalloproteinases (MMPs) belong to a family of Zn^2+^-binding Ca^2+^-dependent endopeptidases whose essential function is proteolysis of the extracellular matrix (ECM), a process that is required in several cellular processes [18]. To date, 24 human MMPs that have structural similarities have been identified and categorize to soluble-type MMP (MMPs) and membrane-type MMPs (MT-MMPs). All MMPs are synthesized as latent enzymes that are either secreted or membrane-anchored. Their expression profiles and substrate specificities vary. In the early stage of MMP investigation, main study was focused on the cancer metastasis and invasion through ECM degradation. Therefore, MMP inhibitor (MMPI) was expected as candidates of attractive drug for cancer and many researcher screened MMPIs [19,20]. We prepared seven kinds of recombinant MMPs, four kinds of soluble-type MMPs (MMP-2, -3, -7, -8), and three kinds of MT-MMPs (MT1-, MT3-, MT4-MMPs) to screen MMPI. However, unfortunately, there is no MMPI being applicable for clinical use because of their severe side effect such as myalgia or joint pain. Apart from ECM molecules, MMPs act on a whole array of substrates including other proteinases and MMPs, proteinase inhibitors, growth factors, cytokines, cell-surface receptors, and cell-adhesion molecules and regulate many processes, such as cell migration, proliferation, apoptosis, angiogenesis, tumor expansion, and metastasis [21,22,23]. Six MT-MMPs have been identified so far, of which four, MT1-/MMP-14, MT2-/MMP-15, MT3-/MMP-16, and MT5-/MMP-24, have a transmembrane domain while the other two, MT4/MMP-17 and MT6-/MMP-25, have a glycosylphosphatidylinositol domain [21]. Among MT-MMPs, MT1-MMP is thought to be a most important in cancer metastasis, because MT1-MMP activates MMP-2, which degrades ECM [24].

In the relationship between PrP, protease K is well known to be the specific enzyme to differ PrP^C^ from a pathogenic or PrP^Sc^ derived from PrP^C^ [25,26]. Recently, Altmeppen *et al.* reviewed the roles of endoproteolytic a-cleavage and shedding of PrP in neurodegeneration [27]. Some a disintegrin and metalloproteinase domain-containing proteins (ADAMs) such as ADAM10, ADAM8, and ADAM17 are thought to contribute to a-cleavage of PrP [28,29,30,31]. In addition, ADAM10 and ADAM9 are thought to contribute to shedding of PrP [32,33,34]. Moreover, the relation ship between MMPs and pathological conditions of the central nervous system is reported [35,36,37]. These lead us to the idea that MMPs may be one of candidate enzyme for PrP digestion.

In another aspect of PrP study, many researchers reported the metal-binding ability of PrP to a divalent metal ion such as Cu^2+^, Ni^2+^, Mn^2+^, *etc.* [38]. Although PrP metal-binding sites have been investigated using full-length PrP^C^ or synthetic fragment peptides and it is now generally accepted that PrP^C^ binds copper *in vivo* [39], most researchers have focused on the octarepeat region [40,41,42,43,44], and there are a few reports describing the metal-binding ability of the middle- and C-terminal domains of hPrP [45]. The interaction of full-length and truncated forms of PrP with Cu^2+^ has been investigated using a range of techniques including electron paramagnetic resonance [46,47,48], circular dichroism (CD) [49,50], X-ray crystallography [51], nuclear magnetic resonance [52,53], mass spectrometry (MS) [54,55,56], Raman spectroscopy [57,58], Fourier transform infrared spectroscopy [59], and potentiometry [60]. Although these methods have been useful, most can only detect metal binding that occurs during conformational conversion and cannot detect metal binding occurring without conformational change. Only MS is capable of detecting metal binding that does not occur in conjunction with conformational conversion. Electrospray ionization (ESI)-MS has the advantage of being able to directly provide speciation information, and has been used to analyze Cu^2+^ binding to hPrP octarepeat peptides [61,62]. In addition, it is very interesting for us, NMR study carried out to determine the location and properties of metal-binding sites on the PrP [52]. We reported the metal-binding abilities of synthetic fragment peptides covered from PrP60-230 to various divalent metal ions by a column switch (CS)-high-performance liquid chromatography (HPLC) system [63,64]. The CS-HPLC can be detectable the metal-binding ability without conformational conversion, and also is very convenient method to avoid the contamination of metal ions which are serious obstacle for MS analysis. The metal-binding abilities of OP-repeat region to Cu^2+^, Ni^2+^ and Co^2+^ were very high with same degree, but Mn^2+^, Ca^2+^, Cd^2+^, Hg^2+^ and Al^3+^ did not bind. The Cu^2+^-binding abilities of the central region were higher than another ions such as Zn^2+^, Ni^2+^ and Co^2+^. The C-terminus region peptides showed the lower metal-binding ability comparison of the central region peptides. The data obtained using this method agreed well with previous CD analyses [38] and NMR study [52,53].

Our final goal of PrP study is not only to identify the critical part of PrP that contribute to acquire the enzyme resistance and aggregation but also to find inhibitor against neurotoxic diseases. We though that synthetic fragment peptides might be more useful and powerful toll than full-length PrP^C^. Thus, in this study, we focused on the MT-MMPs such as MT1-MMP and MT3-MMP that are expressed in the brain as PrP^C^-degrading protease and on Cu^2+^. The digestion of 21 prion fragment peptides by MT1-MMP, MT3-MMP, MMP-7, and human serum (HS) in the presence or absence of Cu^2+^ was analyzed by HPLC, and the cleavage sites were determined by LC-ESI-MS.

## 2. Results and Discussion

### 2.1. Preparation of Synthetic Fragment Peptides

The mature prion protein is composed of two structurally divergent parts, each of them making up roughly one half of the full-length prion protein: the less-structured N-terminal part with its flexible N-terminus and the compact and globular C-terminal domain (Figure 1).

We divided hPrP residues 60–230 into the following three domains in this study: hPrP60–91 as the octapeptide-repeat region (OP-repeat), hPrP92–168 as the central domain containing the neurotoxic domain and a hydrophobic core, and hPrP169–230 as the C-terminal domain containing a disulfate bridge (between amino acids 178 and 213) and two variably occupied N-glycosylation sites (amino acids 180 and 196). The amino-acid sequences of synthetic peptides were selected on the basis of the position of histidine (H) residues and the secondary structure of hPrP [11]. Synthetic fragment peptides of various lengths covered hPrP residues 60–230. The synthetic ratio appeared to be very high, and the purity of the synthetic peptide was sufficient for use in this study. All peptides were characterized by ESI-MS using a direct-spray method.

The OP-repeat region, composed of four highly conserved, contiguous repeats of the eight-residue sequence PHGGGWGQ, is located in the flexible N-terminal region of hPrP. This domain also contains the copper-binding sites. Four peptides corresponding to one to four octarepeats, and the peptide PAGGGWGQ containing an H residue to a residue substitution, were synthesized. We also synthesized an additional eight fragment peptides corresponding to the middle domain of hPrP (amino acids 92–168), each of which contained one to four H residues. Five H-containing peptides and three non-H-containing peptides corresponding to the C-terminal domain of hPrP (amino acids 169–230) were synthesized and characterized in the same manner. The amino acid sequences of the synthetic peptides examined in this study, apart from the OP-region, are described in Table 1.

### 2.2. Determination of Cleavage Sites

**Figure 2:**
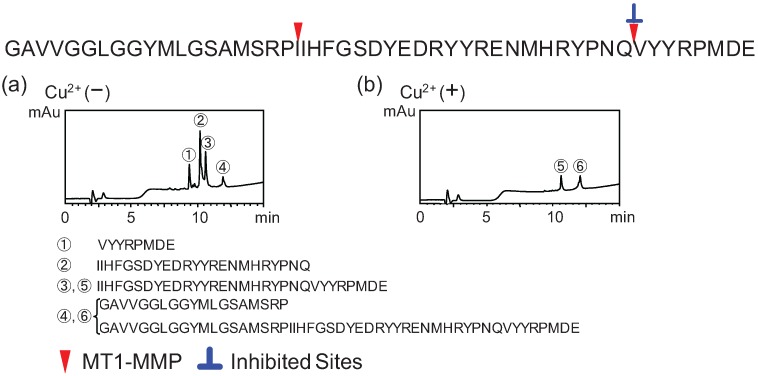
Determination of the cleavage sites on hPrP119–168 by MT1-MMP, and inhibitory effects of Cu^2+^. Reversed-phase HPLC revealed four peaks (Peaks-1 to -4) and two peaks (Peaks-5 and -6), respectively, from reaction mixtures in the absence (**a**) and presence (**b**) of Cu^2+^. Sequence analysis of each peak was performed by direct-injection ESI-MS.

Before carrying out this experiment, we tested the inhibitory activities of Cu^2+^ against MMPs, and confirmed that Cu^2+^ did not show any effects to MMPs in the concentration used here. To study the cleavage specificity of MT1-MMP, MT3-MMP, MMP-7, and HS, each purified peptide was incubated by purified recombinant MMPs (r-MMPs) independently, and products were analyzed by HPLC. As shown in Figure 2a, hPrP119-168, derived from the central region, was digested by MT1-MMP, and four peaks (Peak-1 to Peak-4) were observed. On the other hand, two peaks (Peak-5 and Peak-6) were observed on addition of Cu^2+^. This data indicates that proteolysis of hPrP119-168 by MT1-MMP is partially inhibited by Cu^2+^. In addition, remaining of peaks-5 and -6 proves that Cu^2+^ does not inhibit MT-1 MMP activity in this condition.

**Table 1:** Amino acid sequences of hPrP fragment peptides.

Name	Sequence
60–91(OP-4)	PHGGGWGQPHGGGWGQPHGGGWGQPHGGGWGQ
60–83(OP-3)	PHGGGWGQPHGGGWGQPHGGGWGQ
60–75(OP-2)	PHGGGWGQPHGGGWGQ
60–67(OP–1)	PHGGGWGQ
OP-1/HA	PAGGGWGQ
92–168	GGGTHSQWNKPSKPKTNMKHMAGAAAAGAVVGGLGGYMLGSAMSRPIIHFGSDYEDRYYRENMHRYPNQVYYRPMDE
119–168	GAVVGGLGGYMLGSAMSRPIIHFGSDYEDRYYRENMHRYPNQVYYRPMDE
134–168	MSRPIIHFGSDYEDRYYRENMHRYPNQVYYRPMDE
148–168	RYYRENMHRYPNQVYYRPMDE
92–106	GGGTHSQWNKPSKPK
107–119	TNMKHMAGAAAAG
132–144	SAMSRPIIHFGSD
150–159	YRENMHRYPN
169–192	YSNQNNFVHDCVNITIKQHTVTTT
169–179	YSNQNNFVHDC
169–178	YSNQNNFVHD
175–189	FVHDCVNITIKQHTV
180–192	VNITIKQHTVTTT
180–192/HA	VNITIKQATVTTT
193–230	TKGENFTETDVKMMERVVEQMCITQYERESQAYYQRGS
215–230	ITQYERESQAYYQRGS

To determine the cleavage site, the peaks (Peak-1 to -6) illustrated in Figure 2 were collected and analyzed by ESI-MS with direct injection. Peaks-1 and -2 were identified as hPrP161–168 and hPrP138–160, respectively. Peaks-3 and -5 were identified as hPrP138–168. Peak-4 and -6 were identified as a mixture of hPrP119–137 and hPrP119–168. Thus, we conclude that MT1-MMP digests PrP fragment peptide, the Cu^2+^-binding peptides acquires the MT1-MMP resistance.

**Figure 3:**
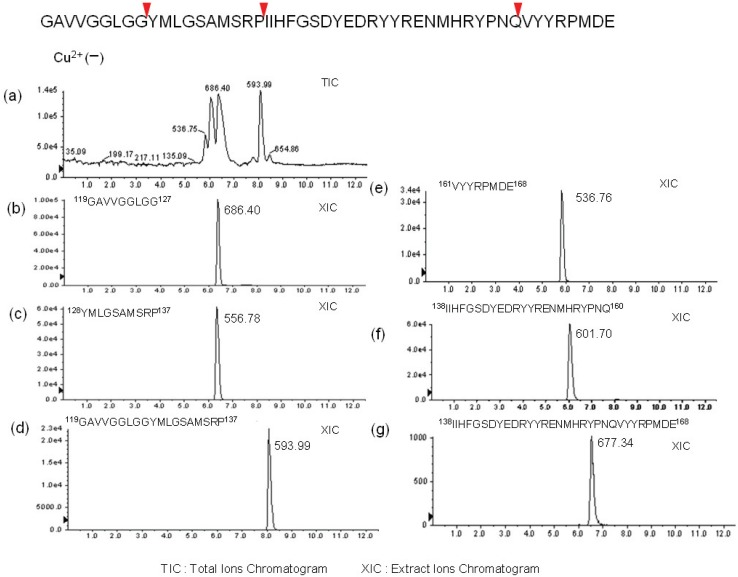
Sequence analysis by LC-ESI-QTOF MS of fragment peptides in the reaction mixture of hPrP119–168 in the absence of Cu^2+^. (**a**) Total ion chromatogram (TIC) of reaction mixture. Extracted ion chromatogram monitored by hPrP119–127 fragment ion (**b**), by hPrP128–137 fragment ion (**c**), by hPrP119–137 fragment ion (**d**), by hPrP161–168 fragment ion (**e**), by hPrP138-160 fragment ion (**f**), by hPrP138-168 fragment ion (**g**).

**Figure 4:**
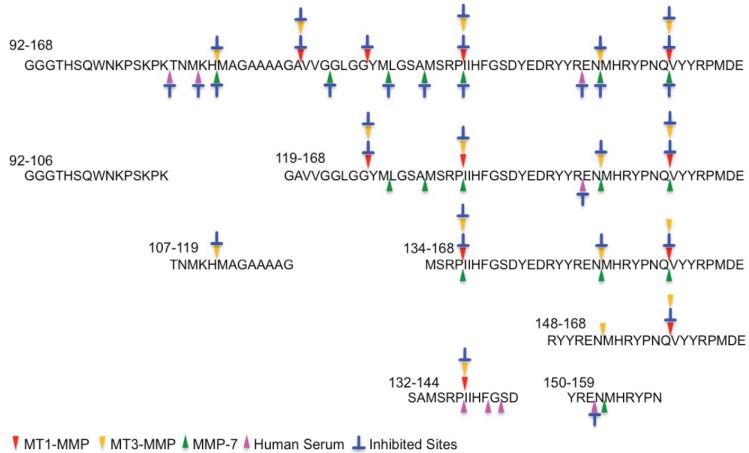
Cleavage sites on the central region peptides. The cleavage sites are marked with colored triangles: MT1-MMP, red arrow; MT3-MMP, yellow arrow; MMP-7, green arrow; HS, pink arrow and the inhibited sites (blue) are indicated.

**Figure 5:**
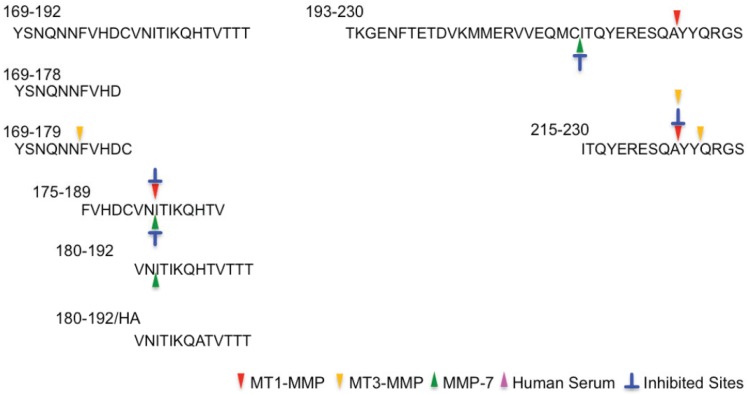
Cleavage sites on the C-terminus region peptides. The cleavage sites are marked with colored triangles: MT1-MMP, red arrow; MT3-MMP, yellow arrow; MMP-7, green arrow; HS, pink arrow and the inhibited sites (blue) are indicated.

To identify partner fragments with identified peaks described in Figure 2, the reaction mixtures were analyzed by LC-ESI-MS. Three major peaks were detected on the total ion chromatogram (Figure 3a; XIC), which were identified as hPrP119–127, hPrP128–137, and hPrP119–137 on the extracted ion chromatograms (Figure 3b–d). In addition, hPrP161–168, hPrP138–160, and hPrP138–168 were also identified as fragment peptide that. On the basis of LC-MS analysis, three cleavage sites, ^127^Gly-Tyr^128^, ^137^Pro-Ile^138^ and ^16^Gln-Val^161^ were additionally identified (Figure 3e–g). The cleavage sites of each peptide by MT1-MMP, MT3-MMP, MMP-7, and HS were determined in the same manner (Figure 4 and Figure 5). These data (Figure 2 and Figure 3) suggests interesting events that.

HPrP119–168 may contain the important amino acid sequence for aggregation of PrP. In comparison with peak heights of each peak in Figure 2, peak-5 is lower than peak-3, it suggests hPrP119–168 acquires the enzyme resistance by binding to Cu^2+^. We think that this event may occur with conformational change with Cu^2+^-binding. In addition, although peak-6 contains hPrP119–168 and hPrP119–137, peak heights of peak-6 is very low. These phenomena suggest that hPrP119–168 may aggregate by Cu^2+^-binding. However, unfortunately, the Cu^2+^-binding peptide was not identified in the acidic conditions used in this LC-MS analysis.

In the case of the OP-repeat region, no peptide was digested by the MMPs tested here. The cleavage sites in the central and C-terminal regions are described in Figure 4 and Figure 5, respectively. In the central region, many cleavage sites were identified. hPrP92–168, the longest fragment peptide in the central region, was digested with MMPs and HS. Four, five and seven cleavage sites were identified by MT1-MMP, MT3-MMP, and MMP-7, respectively: these digestions were inhibited in the presence of Cu^2+^. Three cleavage sites were identified with HS. Interestingly, the cleavage sites of hPrP119–168 and hPrP134–168 were almost the same as those of hPrP92–168, but the inhibitory effect of Cu^2+^ on MMP-7 activity was completely different with hPrP119–168 and hPrP134–168 from that with hPrP92–168. hPrP148–168 was digested with MT1-MMP and MT3-MMP, but not with MMP-7 and HS. hPrP150–159 was digested with MMP-7 and HS, but not with MT3-MMP. hPrP132–144 was digested with HS, despite there being no HS cleavage site in hPrP92-168. In the case of hPrP107–119, only one cleavage site, by MT3-MMP, was identified: the cleavage sites found with MMP-7 and HS in hPrP92–168 were not present. The N-terminus fragment peptide of hPrP92–168, hPrP92–106, was not digested with MMPs or HS. In comparison with all data described in Figure 4, the cleavage sites by MT1-MMP or MT3-MMP, and those inhibitory effects with Cu^2+^ were very similar, although the cleavage sites by MMP-7 were different from MT1-MMP or MT3-MMP. In addition, the inhibitory pattern with Cu^2+^ of hPrP92–168 is completely different from that of hPrP119–168 and hPrP134–168. These differences may be occurred from the conformation of Cu^2+^-binding fragment peptide.

In the case of the C-terminus region, this region forms β-sheet structure in nature PrP^C^ [11], there were a few cleavage sites. hPrP193–230 was cleaved by MT1-MMP and MMP-7 between ^224^Ala–^225^Tyr and ^214^Cys–^215^Ile, respectively. Cleavage by MMP-7 but not MT1-MMP was inhibited in the presence of Cu^2+^. Interestingly, hPrP215-230, the C-terminal end of hPrP193–230, was cleaved by MT1-MMP, but this cleavage reaction was inhibited in the presence of Cu^2+^. In addition, this peptide was cleaved by MT3-MMP, which was not inhibited in the presence of Cu^2+^. CS-HPLC analysis showed that this fragment peptide binds to Cu^2+^ [64]. CD analysis indicated that the C-terminus fragment peptide takes a β-sheet structure on treatment with Cu^2+^ or in acidic conditions (data not shown). These differences in cleavage pattern and Cu^2+^ inhibitory effects between hPrP215–230 and hPrP193–230 might be attributed to the conformational differences. hPrP169–192 was not digested with any MMPs or HS, but the shorter fragment peptides, hPrP169–192, hPrP169–179, hPrP175–189, and hPrP180–192, were digested with MMPs. Interestingly, HS did not digest the C-terminal region. In addition, only MT1-MMP digested hPrP193–230 between ^224^Ala–^225^Tyr, at the C-terminal end of hPrP. Other fragment peptides were not digested with MMPs or HS. This study reveals that MMPs digest PrP fragment peptides at many sites, inhibitory effects with Cu^2+^ is related to the conformational change of Cu^2+^-binding peptide but not result in the direct inhibition to MMPs with Cu^2+^.

**Figure 6:**
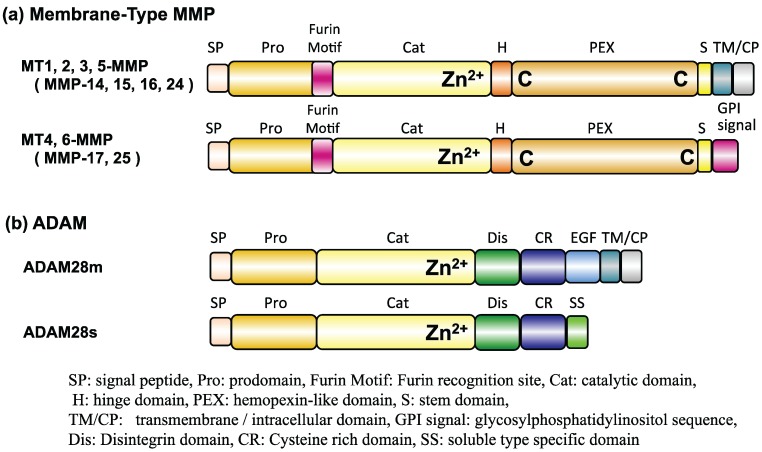
Domain structures of MT-MMPs (**a**) and ADAMs (**b**).

PrP^C^ is subject to diverse proteolytic processing, including a-cleavage within the neurotoxic domain (hPrP105–125 in mice), b-cleavage around amino acid position 90, and shedding near the plasma membrane to release almost full-length PrP^C^ into the extracellular space. ADAMs are thought to be candidate proteinases for PrP^C^ degradation [65]. The domain structure of MMPs is similar to that of ADAMs, and both enzymes are Zn^2+^-dependent metalloproteinases (Figure 6). This study suggests that MMPs, especially MT-MMPs, may act in the physiological processing of PrP^C^.

**Figure 7:**
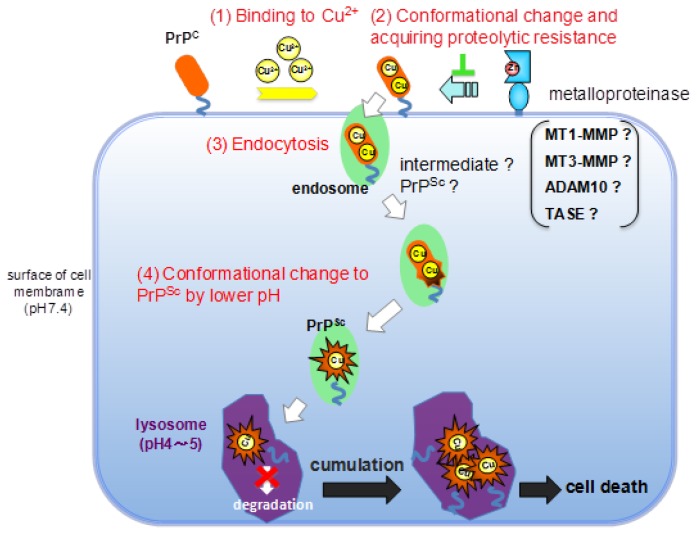
Our hypothesis of the role of membrane-type matrix metalloproteinase and Cu^2+^ for the regulation of PrP^C^. First step (**1**) binding to Cu^2+^; Second step (**2**) conformational change and acquiring proteolytic resistance; Third step (**3**) endocytosis; Fourth step (**4**) conformational change to PrP^Sc^ by lower pH in cell; Final step (**5**) aggregation of PrP^Sc^ and cell death.

Recntly, roles of matrix metalloproteinases and their targets in epileptogenesis and seizures were reviewed by Mizoguchi and Yamada [66]. We here discuss the role of MT-MMPs for PrP homeostasis in cell. Up to date, it is generally accepted that Prion disease occurs in young generation and is caused by the conformational change from PrP^C^ to PrP^Sc^. This conformational change is related to Cu^2+^-binding. The amino acid mutation in PrP^Sc^ is mainly occurred in C-terminus end (C-terminal region in this study) and the central region in next. The fragment peptides in the C-terminus region showed the enzyme resistance and take β-sheet structure with Cu^2+^-binding or in lower pH (data not shown). Thus, we think that the enzyme resistance and aggregation of PrP^Sc^ is caused by conformational change in the C-terminus region. In the case of amino acid mutation, insertion or deletion on OP-repeat region, the homeostasis of Cu^2+^ is in out of order that may promote Cu^2+^-binding to the central and the C-terminus regions. In any case, we think the most important event is the conformational change on the C-terminus region. The data obtained from Prion fragment peptides here support the results reported by many researchers [67,68,69]. We know we are opening ourselves to criticism, but we would like to present here a bold and challenging hypothesis that could be applicable to explain the another neurotoxic diseases, such as Alzheimer’s and Parkinson’s diseases. With increasing age, the concentration of Cu^2+^ in the brain increases by disorder of metabolism [70,71,72], and Zn^2+^ concentration decreases [73]. Once the amount of Cu^2+^ exceeds the control range of the OP-repeat region, which controls Cu^2+^ homeostasis, sites in PrP^C^ can capture the excess Cu^2+^ and acquire resistance against cellular proteinase with a structural change from α-helix to β-sheet. In addition, the proteolytic activity of MMPs is weakened by the low concentration of Zn^2+^. As a result, the full-length PrP^C^ could be taken into the cell via endocytosis and the structure could change to PrP^Sc^, allowing PrP to aggregate in the cell (Figure 7).

## 3. Experimental

### 3.1. Preparation of Recombinant Human MMPs

Recombinant human MT1-MMP, MT3-MMP and MMP-7 (matrilysin) were prepared as described previously [19,74]. In brief, the cDNAs for procatalytic domains of human MT1-MMP, MT3-MMP and MMP-7 were prepared by polymerase chain reactions using sets of primers (5' primer, GGCGGATCCATGCTCGCCTCCCTCGGCTCG, 3' primer, GCCGTCGACGTTCCCGTCACAGATGTTGGG; 5' primer GGCGGATCCATGATTTTATGTGCTACAGTCTGC, 3' primer GCCGTCGACCCCATCACAGATGTTGGGTTTGGC; and 5' primer, GGCGGATCCATGCTGCCGCTGCCTCAGGAG, 3' primer, GCCGTCGACTTTCTTTCTTGAATTACTTCT; respectively) based on the reported sequences, and the templates (pSG-GelA having a 3.3 Kb cDNA fragment of MMP-2, poly(A)+ RNA isolated from a human rectal carcinoma cell line [CaR-1], and pME18S-MT-MMPs having a 3.5 Kb cDNA fragment of MT1-MMP, respectively). The resulting PCR fragments were inserted into the bacterial expression plasmid pTH-72, having a tandem repeat of the T7 promoter and a hexahistidine-Tag-encoding sequence. The expression, purification, and refolding of the human recombinant MMPs were performed as follows: human recombinant proMMPs were produced in *E. coli* strain BL21 (DE3) transfected with the corresponding expression plasmids derived from pTH-72, solubilized in 8 M urea/10 mM Tris-HCl (pH 8.0)/100 mM Na-phosphate/100 mM β-mercaptoethanol, purified with Ni-NTA resin (QIAGEN Inc., Valencia, CA, USA), and refolded by reducing the urea concentration.

### 3.2. Materials

Trifluoroacetic acid (TFA) (Peptide Synthesis Grade), diethyl ether, acetonitrile (CH_3_CN, HPLC grade), 2-amino*-*2-hydroxymethyl-1,3-propanediol (Tris), HgCl_2_, ZnCl_2_, MnCl_2_·4H_2_O, CaCl_2_·2H_2_O, and CoCl_2_·6H_2_O were purchased from Wako Pure Chemical Industries, Ltd., Inc. (Osaka, Japan). Thioanisole, CuCl_2_·2H_2_O, CdCl_2_·2 1/2H_2_O, and NiCl_2_·6H_2_O were obtained from Nacalai Tesque (Kyoto, Japan). 1,2-ethanedithiol and AlCl_3_·6H_2_O were obtained from Kanto Chemical Co., Inc. (Osaka, Japan). Ethylenediaminetetraacetic acid disodium salt dihydrate (EDTA-2Na) and HCl were purchased from Kishida Chemical Co., Ltd. (Osaka, Japan). Piperidine was obtained from Sigma-Aldrich Japan (Tokyo, Japan). *O*-(7-Azabenzo-triazol-1-yl)-*N*,*N*,*N*′,*N*′-tetra-methyluronium hexafluorophosphate (HATU), *N*,*N*-dimethylformamide, 2.0 M *N*,*N*-diisopropylethylamine/*N*-methylpyrrolidone (DIEA), dichloromethane, and *N*-methylpyrrolidone, fluorenylmethoxycarbonyl (Fmoc)-l-amino acid preloaded resin were purchased from Applied Biosystems (ABI, div. Perkin Elmer, Bedford, MA, USA). Fmoc-l-amino acid was obtained from ABI, AAPPTec, LLC (Louisville, KY, USA) and Peptide Institute, Inc. (Osaka, Japan). Milli-Q water was used for all experiments.

### 3.3. Preparation of Synthetic Fragment Peptide

Fragment peptides corresponding to amino acid sequences from human prion protein were synthesized using an automated ABI model 433A peptide synthesizer (0.1 mmoL scale with preloaded resin) from Fmoc-protected l-amino acid derivatives purchased from ABI [63]. After deprotection according to the manufacturer’s protocol, each peptide was purified using reversed-phase HPLC (Capcell Pak C18 column, SG, 10 or 15 mm i.d. × 250 mm; Shiseido Co., Ltd., Tokyo, Japan) with a linear gradient elution from 0.1% TFA to 50% or 70% CH_3_CN containing 0.1% TFA over 30 min. The primary peak was collected and then lyophilized. Peptide purity was confirmed using an analytical HPLC system as described below. Each purified peptide was characterized by ESI-MS using a Qstar Elite Hybrid LC/MS/MS system (ABI, Framingham, MA, USA).

### 3.4. Analytical HPLC

Cleavage reactions and the purity of synthetic peptides were confirmed by analytical reversed-phase HPLC (Cosmosil 5C_18_-AR-II column, 5 mm, 4.6 mm i.d. × 150 mm; Nacalai Tesque, (Kyoto, Japan) with a flow rate of 1.0 mL/min and a linear gradient elution from 0.1% TFA to 70% CH_3_CN containing 0.1% TFA over 15 min. The column eluate was monitored with a photodiode-array detector (SPD-M20A; Shimadzu, Kyoto, Japan).

### 3.5. Determination of Cleavage Sites

Each purified protein fragment peptide (final conc. 0.1 mM) was individually incubated with recombinant MT1-MMP or MT3-MMP in the presence or absence of Cu^2+^ (two equivalent molecule of His residue in each peptide; final conc. 0 to 0.8 mM) at 37 °C for 1 day in assay buffer (final conc. 50 mM Tris-HCl (pH 7.5), 150 mM NaCl, 10 mM CaCl_2_, 5 mM ZnCl_2_, 0.005% Briji 35, 3 mM NaN_3_). After boiling for 5 min, the reaction mixtures were analyzed by analytical HPLC monitored with a photodiode-array UV detector. The determination of cleavage sites was performed by ESI-MS and LC-ESI-MS analysis using a Qstar Elite Hybrid LC/MS/MS system (ABI). Recombinant MMP-7 and HS were also tested as control.

## 4. Conclusions

In this study, we found that MT1-MMP and MT3-MMP degrade hPrP fragment peptides with many cleavage sites, and that these activities were inhibited by treatment with Cu^2+^. hPrP61–90 from the OP-repeat region was cleaved by HS, but showed protease resistance against MMPs. On the other hand, hPrP92–168 from the central region was cleaved by MT1-MMP, MT3-MMP and MMP-7. These cleavage reactions were inhibited by treatment with Cu^2+^. The C-terminal peptides possessed higher resistance than the central region. hPrP193–230, the longest fragment peptide of the C-terminus, was cleaved by MT1-MMP and MMP-7. Interestingly, cleavage by MT1-MMP was not inhibited by treatment with Cu^2+^, unlike cleavage by MMP-7. These data indicate the possibility that MT1-MMP might shed hPrP in a similar way to ADAMs. Although we are now on the half way to our final goal, the data obtained from this study may be useful to open the next door of PrP research.

## References

[1] Prusiner S.B. (1991). Molecular biology of prion diseases. Science.

[2] Chazot G., Broussolle E., Lapras C., Blattler T., Aguzzi A., Kopp N. (1996). New variant of Creutzfeldt-Jakob disease in a 26-year-old French man. Lancet.

[3] Will R.G., Ironside J.W., Zeidler M., Cousens S.N., Estibeiro K., Alperovitch A., Poser S., Pocchiari M., Hofman A., Smith P.G. (1996). A new variant of Creutzfeldt-Jakob disease in the UK. Lancet.

[4] Prusiner S.B. (1997). Prion diseases and the BSE crisis. Science.

[5] Pan K.M., Baldwin M., Nguyen J., Gasset M., Serban A., Groth D., Mehlhorn I., Huang Z., Fletterick R.J., Cohen F.E. (1993). Conversion of α-helices into beta-sheets features in the formation of the scrapie prion proteins. Proc. Natl. Acad. Sci. USA.

[6] Safar J., Roller P.P., Gajdusek D.C., Gibbs C.J. (1993). Conformational transitions, dissociation, and unfolding of scrapie amyloid (prion) protein. J. Biol. Chem..

[7] Cohen F.E., Pan K.M., Huang Z., Baldwin M., Fletterick R.J., Prusiner S.B. (1994). Structural clues to prion replication. Science.

[8] Telling G.C., Scott M., Mastrianni J., Gabizon R., Torchia M., Cohen F.E., DeArmond J., Prusiner S.B. (1995). Prion propagation in mice expressing human and chimeric PrP transgenes implicates the interaction of cellular PrP with another protein. Cell.

[9] Kaneko K., Zulianello L., Scott M., Cooper C.M., Wallace A.C., James T.L., Cohen F.E., Prusiner S.B. (1997). Evidence for protein X binding to a discontinuous epitope on the cellular prion protein during scrapie prion propagation. Proc. Natl. Acad. Sci. USA.

[10] Harris D.A., Huber M.T., van Dijken P., Shyng S.L., Chait B.T., Wang R. (1993). Processing of a cellular prion protein: Identification of N- and C-terminal cleavage sites. Biochemistry.

[11] Zahn R., Liu A., Luhrs T., Riek R., von Schroetter C., Lopez Garcia F., Billeter M., Calzolai L., Wider G., Wuthrich K. (2000). NMR solution structure of the human prion protein. Proc. Natl. Acad. Sci. USA.

[12] Stahl N., Borchelt D.R., Hsiao K., Prusiner S.B. (1987). Scrapie prion protein contains a phosphatidylinositol glycolipid. Cell.

[13] Forloni G., Angeretti N., Chiesa R., Monzani E., Salmona M., Bugiani O., Tagliavini F. (1993). Neurotoxicity of a prion protein fragment. Nature.

[14] Selvaggini C., de Gioia L., Cantu L., Ghibaudi E., Diomede L., Passerini F., Forloni G., Bugiani O., Tagliavini F., Salmona M. (1993). Molecular characteristics of a protease-resistant, amyloidogenic and neurotoxic peptide homologous to residues 106–126 of the prion protein. Biochem. Biophys. Res. Commun..

[15] Brown D.R. (1999). Prion protein peptide neurotoxicity can be mediated by astrocytes. J. Neurochem..

[16] Ettaiche M., Pichot R., Vincent J.P., Chabry J. (2000). *In vivo* cytotoxicity of the prion protein fragment 106–126. J. Biol. Chem..

[17] O’Donovan C.N., Tobin D., Cotter T.G. (2001). Prion protein fragment PrP-(106–126) induces apoptosis via mitochondrial disruption in human neuronal SH-SY5Y cells. J. Biol. Chem..

[18] Murphy G., Nagase H. (2011). Localization matrix metalloproteinase activities in the pericellular enviroment. FEBS J..

[19] Oku N., Matsukawa M., Yamakawa S., Asai T., Yahara S., Hashimoto F., Akizawa T. (2003). Inhibitory effect of green tea polyphenols on membrane-type 1 matrix metalloproteinase, MT1-MMP. Biol. Pharm. Bull..

[20] Millar A.W., Brown P.D., Moore J., Galloway W.A., Cornish A.G., Lenehan T.J., Lynch K.P. (1998). Results of single and repeat dose studies of the oral matrix metalloproteinase inhibitor marimastat in healthy male volunteers. Br. J. Clin. Pharmacol..

[21] Zitka O., Kukacka J., Krizkova S., Huska D., Adam V., Masarik M., Prusa R., Kizek R. (2010). Matrix metalloproteinases. Curr. Med. Chem..

[22] Butler G.S., Overall C.M. (2009). Updated biological roles for matrix metalloproteinases and new “intracellular” substrates revealed by degradomics. Biochemistry.

[23] Rodriguez D., Morrison J., Overall C.M. (2010). Matrix metalloproteinases: What do they not do? New substrates and biological roles identified by murine models and proteomics. Biochim. Biophys. Acta.

[24] Sato H., Takino T., Okada Y., Cao J., Shinagawa A., Yamamoto E., Seiki M. (1994). A matrix metalloproteinase expressed on the surface of invasive tumour cells. Nature.

[25] Prusiner S.B., Scott M.R., DeArmond S.J., Cohen F.E. (1998). Prion protein biology. Cell.

[26] Oesch B., Westaway D., Wälchli M., McKinley M.P., Kent S.B., Aebersold R., Barry R.A., Tempst P., Teplow D.B., Hood L.E. (1985). A cellular gene encodes scrapie PrP 27–30 protein. Cell.

[27] Altmeppen H.C., Prox J., Puig B., Dohler F., Falker C., Krasemann S., Glatzel M. (2013). Roles of endoproteolytic a-cleavage and shedding of the prion protein in neurodegeration. FEBS J..

[28] Vincent B., Paitel E., Saftig P., Frobert Y., Hartmann D., de Strooper B., Grassi J., Lopez-Perez E., Checler F. (2001). The disintegrins ADAN10 and TACE contribute to the constitutive and phorbol ester-regulated normal cleavage of the cedllular prion protein. J. Biol. Chem..

[29] Liang J., Wang W., Sorensen D., Medina S., Ilchenko S., Kiselar J., Surewicz W.K., Booth S.A., Kong Q. (2012). Cellular prion protein regulates ist own alpha-cleabage through ADAM8 in skeletal muscle. J. Biol. Chem..

[30] Beland M., Motard J., Barbarin A., Roucou X. (2012). PrP(C) homodimerization stimulates the production of PrPC cleaved fragments PrPN1 and PrPC1. J. Neurosci..

[31] Wik L., Klingeborn M., Willander H., Linner T. (2012). Separate mechanisms act concurrently to shed and release the prion protein from the cell. Prion.

[32] Cisse M.A., Sunyach C., Lefrance-Jullien S., Postina R., Vincent B., Checler F. (2005). The disintegrin ADAM9 indirectly contributes to the physiological processing of cellular prion by modulating ADAM10 activity. J. Biol. Chem..

[33] Tousseyn T., Thathiah A., Jorissen E., Snellinx A., Serneels L., Nyabi O., Annaert W., Saftig P., Hartmann D., de Strooper B. (2009). ADAM10, the rate-limiting protease of regulated intramembrane proteolysis of Notch and other proteins, is processed by ADAMS-9, ADAMS-15, and the gamma-secretase. J. Biol. Chem..

[34] Moss M.L., Powell G., Miller M.A., Edwards L., Qi B., Sang Q.X., de Strooper B., Tesseur I., Lichtenthaler S.F., Taverna M. (2011). ADAM9 inhibition increases membrane activity of ADAM10 and controls alpha-secretase processing of amyloid precursor protein. J. Biol. Chem..

[35] Yong V.W., Power C., Forsyth P., Edwards D.R. (2001). Metalloproteinases in biology and pathology of the nervous system. Nat. Rev. Neurosci..

[36] Mizoguchi H., Ibi D., Takuma K., Toth E., Sato J., Itohara S., Nabeshima T., Yamada K. (2010). Alterations of emotional and cognitive behaviors in matrix metalloproteinase-2 and -9-deficient mice. Open Behav. Sci. J..

[37] Mizoguchi H., Yamada K., Nabeshima T. (2011). Matrix metalloproteinases contribute to neuronal dysfunction in animal models of drug dependence, Alzheimer’s disease, and epilepsy. Biochem. Res. Int..

[38] Stöckel J., Yamada K., Safar J., Wallace A.C., Cohen F.E., Prusiner S.B. (1998). Prion protein selectively binds copper(II) Ions. Biochemistry.

[39] Brown D.R., Qin K., Herms J.W., Madlung A., Manson J., Strome R., Fraser P.E., Kruck T., von Bohlen A., Schulz-Schaeffer W. (1997). The cellular prion protein binds copper *in vivo*. Nature.

[40] Miura T., Hori-i A., Takeuchi H. (1996). Metal-dependent alpha-helix formation promoted by the glycine-rich octapeptide region of prion protein. FEBS Lett..

[41] Walter E.D., Chattopadhyay M., Millhauser G.L. (2006). The affinity of copper binding to the prion protein octarepeat domain: Evidence for negative cooperativity. Biochemistry.

[42] Wells M.A., Jackson G.S., Jones S., Hosszu L.L., Craven C.J., Clarke A.R., Collinge J., Waltho J.P. (2006). A reassessment of copper (II) binding in the full-length prion protein. Biochem. J..

[43] Wells M.A., Jelinska C., Hosszu L.L., Craven C.J., Clarke A.R., Collinge J., Waltho J.P., Jackson G.S. (2006). Multiple forms of copper (II) co-ordination occur throughout the disordered N-terminal region of the prion protein at pH 7.4. Biochem. J..

[44] Klewpatinond M., Davies P., Bowen S., Brown D.R., Viles J.H. (2008). Deconvoluting the Cu^2+^ binding modes of full-length prion protein. J. Biol. Chem..

[45] Ronga L., Palladino P., Saviano G., Tancredi T., Benedetti E., Ragone R., Rossi F. (2007). NMR structure and CD titration with metal cations of human prion alpha2-helix-related peptides. Bioinorg. Chem. Appl..

[46] Aronoff-Spencer E., Burns C.S., Avdievich N.I., Gerfen G.J., Peisach J., Antholine W.E., Ball H.L., Cohen F.E., Prusiner S.B., Millhauser G.L. (2000). Identification of the Cu^2+^ binding sites in the N-terminal domain of the prion protein by EPR and CD spectroscopy. Biochemistry.

[47] Burns C.S., Aronoff-Spencer E., Legname G., Prusiner S.B., Antholine W.E., Gerfen G.L., Peisach J., Millhauser G.L. (2003). Copper coordination in the full-length, recombinant prion protein. Biochemistry.

[48] Bonomo R.P., Imperllizzeri G., Pappalardo G., Rizzarelli E., Tabbi G. (2000). Copper(II) binding modes in the prion octapeptide PHGGGWGQ: A spectroscopic and voltammetric study. Chemistry.

[49] Viles J.H., Cohen F.E., Prusiner S.B., Goodin D.B., Wright P.E., Dyson H.J. (1999). Copper binding to the prion protein: Structural implications of four identical cooperative binding sites. Proc. Natl. Acad. Sci. USA.

[50] Hornshaw M.P., McDermott J.R., Candy J.M., Lakey J.H. (1995). Copper binding to the N-terminal tandem repeat region of mammalian and avian prion protein: Structural studies using synthetic peptides. Biochem. Biophys. Res. Commun..

[51] Burns C.S., Aronoff-Spencer E., Dunham C.M., Lario P., Avdievich N.I., Antholine W.E., Olmstead M.M., Vrielink A., Gerfen G.L., Peisach J. (2002). Molecular features of the copper binding sites in the octarepeat domain of the prion protein. Biochemistry.

[52] Jackson G.S., Murray I., Hosszu L.L.P., Gibbs N., Walltho J.P., Clarke A.R., Collinge J. (2001). Location and properties of metal-binding sites on the human prion protein. Proc. Natl. Acad. Sci. USA.

[53] Jones C.E., Klewpatinond M., Abdelraheim S.R., Brown D.R., Viles J.H. (2005). Probing copper^2+^ binding to the prion protein using diamagnetic nickel^2+^ and ^1^H NMR: The unstructured N terminus facilitates the coordination of six copper^2+^ ions at physiological concentrations. J. Mol. Biol..

[54] Qin K.F., Yang Y., Mastrangelo P., Westaway D. (2002). Mapping Cu(II) binding sites in prion proteins by diethyl pyrocarbonate modification and matrix-assisted laser desorption ionization-time of flight (MALDI-TOF) mass spectromeric footprinting. J. Biol. Chem..

[55] Whittal R.M., Ball H.L., Cohen F.E., Burlingame A.L., Prusiner S.B., Baldwin M.A. (2000). Copper binding to octarepeat peptides of the prion protein monitored by mass spectrometry. Protein Sci..

[56] Hornshaw M.P., McDermott J.R., Candy J.M. (1995). Copper binding to the N-terminal tandem repeat regions of mammalian and avian prion protein. Biochem. Biophys. Res. Commun..

[57] Miura T., Hori-i A., Mototani H., Takeuchi H. (1999). Raman spectroscopic study on the copper(II) binding mode of prion octapeptide and its pH dependence. Biochemistry.

[58] Miura T., Sasaki S., Toyama A., Takeuch H. (2005). Copper reduction by the octapeptide repeat region of prion protein: pH dependence and implicatios in cellular copper uptake. Biochemistry.

[59] Gustiananda M., Haris P.I., Milburm P.J., Gready J.E. (2002). Copper-induced conformational change in a marsupial prion protein repeat peptide probed using FTIR spectroscopy. FEBS Lett..

[60] Valensin D., Luczkozlowski M., Mancini F.M., Legowska A., Gaggelli E., Valensin G., Rolka K., Kozlowski H. (2004). The dimeric and tetrameric octarepeat fragments of prion protein behave differently to its monomeric unit. Dalton Trans..

[61] Yu X., Wojciechowski M., Fenselau C. (1993). Assessment of metals in reconstituted metallothioneins by electrospray mass spectrometry. Anal. Chem..

[62] Lim J., Vachet R.W. (2004). Using mass spectrometry to study copper-protein binding under native and non-native conditions: Beta-2-microglobulin. Anal. Chem..

[63] Kojima A., Konishi M., Akizawa T. (2010). Metal-binding ability of peptides originating from prion protein by a column switch HPLC. Pept. Sci..

[64] Kojima A., Mabuchi Y., Konishi M., Okihara R., Nagano M., Akizawa T. (2011). Metal-binding ability of human prion protein fragment peptides analyzed by column switch HPLC. Chem. Pharm. Bull..

[65] Altmeppen H.C., Puig B., Dohler F., Thurm D.K., Falker C., Krasemann S., Glatzel M. (2012). Proteolytic processing of the prion in health and disease. Am. Neurodegener. Dis..

[66] Mizoguchi H., Yamada K. (2013). Roles of matrix metalloproteinases and their targets in epileptogenesis and seizures. Clin. Psychopharmacol. Neurosci..

[67] Goldfarb L.G., Brown P., McCombie W.R., Goldgaber D., Swergold G.D., Wills P.R., Cervenakova L., Baron H., Gibbs C.J., Gajdusek D.C. (1991). Transmissible familial Creutzfeldt-Jakob disease associated with five, seven, and eight extra octapeptide coding repeats in the PRNP gene. Proc. Natl. Acad Sci. USA.

[68] Moore R.A., Herzog C., Errett J., Kocisko D.A., Arnold K.M., Hayes S.F., Priola S.A. (2006). Octapeptide repeat insertions increase the rate of protease-resistant prion protein formation. Protein Sci..

[69] Li B., Qing L., Yan J., Kong Q. (2011). Instability of the octarepeat region of the human prion protein gene. PLoS One.

[70] Fell G.S., Smith H., Howie R.A. (1968). Neutron activation analysis OFR copper in biological material applied to Wilson’s disease. J. Clin. Pathol..

[71] Sass-Kortsak A. (1965). Copper metabolism. Adv. Clin. Chem..

[72] Pal A., Kumar A., Prasad R. (2014). Predictive association of copper metabolism proteins with Alzheimer’s disease and Parkinson’s disease: A preliminary perspective. Biometals.

[73] Constantinidis J. (1992). Treatment of Alzheimer’s disease by zinc compounds. Drug Dev. Res..

[74] Itoh M., Masuda K., Ito Y., Akizawa T., Yoshioka M., Imai K., Okada Y., Sato H., Seiki M. (1996). Purification and refolding of recombinant human proMMP-7 (pro-matrilysin) expressed in Escherichia coli and its characterization. J. Biochem..

